# Screening of Solvent Systems for Countercurrent Chromatography Separation of Polar Constituents from *Ginkgo biloba* L. Seeds

**DOI:** 10.3390/molecules30020409

**Published:** 2025-01-19

**Authors:** Ruxi Hu, Zhuo Liu, Yi Zhou, Peng Tian, Luqi Li, Zhi Yang, Yatuan Ma

**Affiliations:** 1 College of Chemistry & Pharmacy, Northwest A&F University, 22 Xiong Road, Yangling 712100, China; hrx041004@nwsuaf.edu.cn (R.H.); liuzzhuo@nwafu.edu.cn (Z.L.); izzhouyi@nwafu.edu.cn (Y.Z.); 2008116619@nwafu.edu.cn (P.T.); 2 Life Science Research Core Services, Northwest A&F University, 22 Xiong Road, Yangling 712100, China; liluqi@nwafu.edu.cn

**Keywords:** countercurrent chromatography, *Ginkgo biloba* L., di-(2-ethylhexyl) phosphoric acid, aqueous two-phase systems, inner-recycling chromatography

## Abstract

The separation of large polar constituents presents a substantial challenge in natural product research when employing column chromatography techniques, as the process is both complex and time-consuming. In this study, an acetonitrile/tetrahydrofuran/di-(2-ethylhexyl) phosphoric acid/aqueous saturated sodium chloride solvent system was developed and utilized for the countercurrent chromatography of polar constituents from *Ginkgo biloba* L. seeds. Five polar constituents were effectively isolated using an acetonitrile/tetrahydrofuran/di-(2-ethylhexyl) phosphoric acid/aqueous saturated sodium chloride (2:2:0.8:3, *v*/*v*) solvent system using a two-step countercurrent chromatography method. In the initial countercurrent chromatography process, three constituents were successfully purified from the methanol extract: compound **1**, compound **4**, and compound **5**. Compounds **2** and **3**, co-eluted from the column, were further subjected to three inner-recycling chromatographic procedures. At last, five constituents were purified and identified, including 4′-O-methylpyridoxine (**1**); two indole alkaloid N-glucosides, ginkgoside B (**2**) and ginkgoside A (**3**); 2-(4-hydroxybenzyl) malic acid (**4**); and coniferyl alcohol (**5**). The results demonstrated that the acetonitrile/tetrahydrofuran/di-(2-ethylhexyl) phosphoric acid/aqueous saturated sodium chloride solvent system serves as a feasible system for the efficient countercurrent chromatography separation of polar components.

## 1. Introduction

Centrifugal countercurrent separation (CCCS) is a separation technique that utilizes centrifugal force to achieve a continuous extraction process based on a biphasic solvent system [1]. The primary apparatuses employed for the CCCS of natural and synthetic products are countercurrent chromatography (CCC) and centrifugal partition chromatography (CPC), with CCC utilizing a variable-gravity field and CPC relying on a constant-gravity field [2]. The liquid–liquid separation properties allow for the prevention of irreversible sample adsorption, while also providing benefits such as low solvent consumption and high solute loading capacity [3,4]. During CCCS, the biphasic solvent system selection plays a pivotal role in achieving effective separation, providing appropriate *K* values for the target compounds [5]. Theoretically, there exists an infinite array of solvent systems for CCCS [5]. The solvent systems utilized by CCC can be similarly employed for CPC. In general, the *S_f_* is higher in CCC compared to CPC; however, this trend is reversed when utilizing aqueous two-phase systems (ATPSs) or highly hydrophilic systems due to the smaller density differences between the phases and increased viscosity [6,7]. However, CCC is more commonly employed due to its enhanced cost-effectiveness. As a result, when utilizing CCC for the separation of polar constituents, it often becomes necessary to develop and optimize instrument construction procedures or solvent systems.

During the CCCS of highly polar components, using an ATPS emerge as a favorable choice. Among various ATPS options, polymer–inorganic salt systems have gained extensive utilization for the efficient separation of proteins and polysaccharides [8,9,10]. Additionally, biphasic systems comprising hydrophilic organic solvents and inorganic salt solutions have been proposed and utilized for the purification of significantly hydrophilic natural products [11,12,13]. Moreover, the incorporation of solvent system modifiers, such as ionic liquids and di-(2-ethylhexyl) phosphoric acid (DEHPA), effectively enhances the partition coefficient of polar components [14,15,16].

*Ginkgo biloba* L. has survived on earth for more than 200 million years [17]. In traditional Chinese medicine (TCM), the seeds and leaves of *G. biloba* are considered medicinal components, and there is historical preference for utilizing the former in treating pulmonary disorders [18]. In recent decades, the utilization and development of *G. biloba* seeds have not received adequate attention compared to the ginkgo leaves and exocarps. Ginkgo leaves and seeds contain a variety of bioactive compounds, including flavonoids, terpenes, and polysaccharides. However, the flavonoid and terpenoid content in the seeds is considerably lower than that found in the ginkgo leaves [19], which may contribute to the relative neglect of seed-based research. For *G. biloba*, a variety of extraction, analysis, and separation techniques have been developed. The use of deep eutectic solvents is emerging as an effective and popular approach for extracting active compounds from both ginkgo biloba leaves and exocarps [20,21]. LC-MS-based analysis offers significant advantages for the chemical profiling, metabolic analysis, and selection of active ingredients in *G. biloba* [22,23,24,25]. To obtain compounds with high purities from *G. biloba*, in addition to commonly used column chromatographic separation techniques such as HPLC and silica gel column chromatography, CCC stands out among various separation techniques for its effective separation of components [26,27,28]. For *G. biloba* seeds, the primary research focus is on developing effective methods to eliminate the toxic compounds they contain [29,30]. Despite its numerous benefits in treating conditions such as bronchitis, stomach pain, asthma, cognitive dysfunction, and tuberculosis [29], there are only a limited number of studies focusing on its small-molecule components. However, recent studies have revealed the presence of a class of polar indole alkaloid N-glycosides with pronounced anti-inflammatory activities and anti-skin-aging effects [31,32]. The purification of these compounds required multiple column separations, highlighting the need for more-efficient separation methods. In this study, we developed and employed an acetonitrile/tetrahydrofuran/di-(2-ethylhexyl) phosphoric acid/aqueous saturated sodium chloride solvent system for separating polar constituents from *G. biloba* seeds. Through a two-step CCC process, five polar compounds, including two indole alkaloid N-glucosides, were successfully purified. This system serves as an effective alternative when an ATPS fails to satisfy the separation requirements for polar natural products.

## 2. Results and Discussion

### 2.1. HPLC Analysis and CCC Solvent System Selection

**Figure 1:**
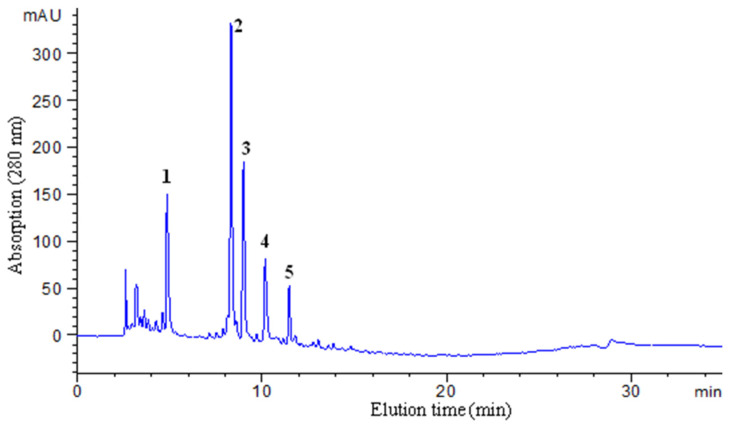
HPLC analysis of the crude extract of *G. biloba* seeds. The five primary components identified in the extract were designated as **1** through **5**.

The primary constituents consist of starch (60–70%), protein (>8%), and ginkgo oils (3.6–7.1%) [19]. Consequently, only approximately 50 g of methanol extract was obtained from 6 kg of fresh seeds. Additionally, the presence of larger particles in their fresh state and extraction mode further impacted the extraction efficiency. Compared to the extensive research that has been conducted on the chemical composition of *G. biloba* leaves, studies focusing on the seeds are notably limited. Therefore, this study initially subjected the seed extract to HPLC analysis. As illustrated in Figure 1, the diversity of components is comparatively lower than that observed in ginkgo leaves [28], with the majority being polar components. In recent studies, Jin Tang Cheng et al. and Yea Jung Choi et al. identified a class of indole alkaloid N-glycosides that exhibit low cytotoxicity, significant anti-inflammatory activities, and potent anti-skin-aging effects [31,32]. However, the purification of these compounds currently requires multiple rounds of column chromatography, which is time-consuming and labor-intensive. Therefore, it is crucial to develop a rapid and efficient separation method to facilitate the enrichment and isolation of these bioactive components. CCC, as a liquid–liquid separation device, plays a crucial role in the separation of complex natural products. High-purity products are typically obtained through a one- or two-step separation process.

During comprehensive CCCS, the selection of a suitable solvent system plays a pivotal role in achieving successful separation, providing a “sweet spot” between 0.4 and 2.5 for the targets [33]. The use of two-phase solvent systems allows one to choose solvents from an enormous number of possible combinations. Without consulting the literature, the search for a two-phase solvent system for the successful separation of particular compounds from a complex sample mixture can be very time-consuming. In order to effectively address the CCCS requirements for polar components, we initially opted for a commonly employed polar solvent system consisting of ethyl acetate, n-butanol, and water. When the ethyl acetate/n-butanol/water (*v*/*v*, 0:1:1) solvent system was utilized to determine the *K* values, only compound **1** exhibited a suitable *K* value of 1.20, whereas compounds **2**–**4** were predominantly distributed in the aqueous phase, and compound **5** was primarily found in the organic phase (shown in Table 1). For ionizable analytes, such as acidic analyte or basic analyte, the introduction of additives is a crucial strategy for tailoring the selectivity of solvent systems to achieve the desired *K* values [34,35]. Therefore, hydrochloric acid and triethylamine were added into the n-butanol/water solvent system, resulting in respective *K* values of 1.30 and 0.31 for compound **1**. The distribution of compounds **2**–**4** and **5** remained undesirable. The determination of *K* values was subsequently conducted using more polar solvent systems, specifically acetonitrile with aqueous saturated NaCl and isopropanol with aqueous saturated NaCl. However, as indicated in Table 1, both solvent systems failed to meet the requirements of CCCS. Furthermore, solvent systems consisting of ethanol or methanol in conjunction with inorganic salts such as NaCl and (NH_4_)_2_SO_4_ in aqueous solutions were also examined. However, these systems were not selected due to the absence of delamination or the occurrence of severe emulsification.

ATPSs possess the distinctive capability to partition into two immiscible aqueous phases, rendering them suitable for the separation of polar components [36]. An ATPS typically comprises a heavier, salt-rich phase and an upper phase enriched with polymers. This method has been widely utilized for the separation of polar constituents, such as proteins and polysaccharides [10,37,38,39]. Therefore, one ATPS of PEG1000/ammonium sulfate/water was used for the determination of *K* values. The results presented in Table 1 demonstrated that both the 15% PEG-1000/15% (NH_4_)_2_SO_4_ and 18% PEG-1000/15% (NH_4_)_2_SO_4_ solvent systems exhibit suitable *K* values for compounds **1–4**, while compound **5** could be effectively separated using an elution–extrusion mode. Consequently, the selection of the 18% PEG-1000/15% (NH_4_)_2_SO_4_ solvent system for *G. biloba* seed extract separation was based on its significantly shorter settling time compared to the alternative option.

### 2.2. CCCS Using an ATPS

**Figure 2:**
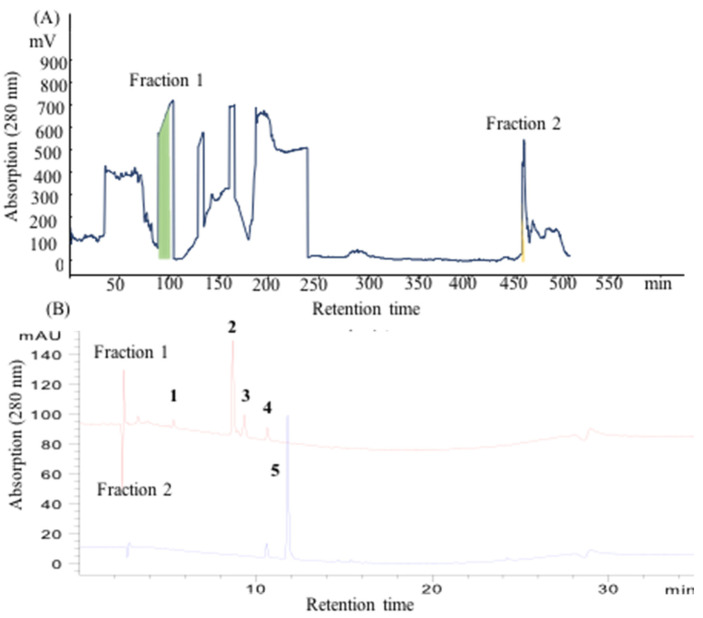
(**A**) CCC profile using the 18% PEG-1000/15% (NH_4_)_2_SO_4_ solvent system. The upper phase is the stationary phase, while the lower phase is the mobile phase. Rotation speed: 1100 rpm, flow rate: 2 mL/min, *S_f_*: 36.9%. (**B**) HPLC analysis of the fractions. The components numbered **1** to **5** represent the five primary elements identified in the extract.

The utilization of an ATPS typically results in a relatively lower *S_f_* for the commonly employed type-J CCC. Enhancing and optimizing the separation column represents an effective approach [40,41], while parameter optimization also serves as a viable method. The achievement of a higher *S_f_* could be attributed to the utilization of parameters such as an elevated rotation speed (1100 rpm) and a reduced flow rate (2 mL/min) [42,43]. Under these experimental conditions, an Sf value of 36.9% was obtained. However, the co-elution of compounds **1**–**4** from the columns occurred, as depicted in Figure 2. Compound **5**, with a HPLC purity of 90%, was separated during the extrusion step. The reason for this might be their similar *K* values. It is noteworthy that a significant disparity existed between the measurement retention time of the sample on the column and its theoretical retention time, and this disparity frequently occurred in acidic or alkaline components. The investigation of solvent systems warranted further exploration in order to achieve the effective separation of the targets.

### 2.3. DEHPA-Modified Solvent System for the CCCS

During CCCS, the utilization of additives serves as a pivotal approach to strategically manipulate the selectivity of either or both phases, thereby facilitating the attainment of desired separation outcomes. The most commonly used metal extractant, DEHPA, was initially employed by Ito et al. as a ligand to enhance the separation of polar catecholamines and dipeptides in CCCS [44,45]. Therefore, the utilization of DEHPA as an adjuvant was employed to tailor the *K* values of components within *G. biloba* seed extract. The solubility of DEHPA in water is extremely low, rendering it unsuitable as a modifier for polymer–inorganic salts or polymer–polymer ATPSs. Consequently, an ATPS employing hydrophilic organic solvents and inorganic salt solutions was employed to develop the solvent system. The introduction of DEHPA into the n-butanol/water solvent system resulted in significant emulsification, which is detrimental to CCC separations. Upon the addition of DEHPA to the ACN/aqueous saturated NaCl solvent system, a tripartite phase separation occurred. To enhance the delamination of the ACN/DEHPA/aqueous saturated NaCl solvent system and reduce the settling time, a series of low-viscosity organic solvents, including methanol, ethanol, and THF, were evaluated. The results indicated that the addition of THF was more suitable for CCCS, as it provided an appropriate up–down phase ratio and significantly shortened the settling time. The ACN/THF/DEHPA/aqueous saturated NaCl solvent system (2:2:x:3, *v*/*v*) was employed for the determination of *K* values, yielding an appropriate volume ratio for the upper and lower phases. The *K* values of the five main constituents in ACN/THF/DEHPA/aqueous saturated NaCl (2:2:x:3, *v*/*v*) solvent systems, as presented in Table 2, all met the separation requirements when operated in an elution–extrusion mode [46,47]. The inclusion of DEHPA significantly enhanced the partitioning of polar constituents into organic phases, as evidenced by the lower distribution of all the five main components in the upper phase in the absence of DEHPA. Therefore, it is plausible that DEHPA acts as a ligand within the organic phase [16,44]. The mechanism may be attributed to the amphiphilic properties of DEHPA, where its phosphoric groups can form hydrogen bonds with analytes. This interaction increases the partitioning of target components into the organic phase, thereby enhancing *K* values and separation efficiency.

The *S_f_* in CCC exhibits a strong correlation with the settling time of the two phases in a test tube [5]. Therefore, the settling times of the ACN/THF/DEHPA/aqueous saturated NaCl (2:2:x:3, *v*/*v*) solvent systems with different ratios of DEHPA were measured. The settling time decreased as the DEHPA ratio increased, as demonstrated in Table 2. Upon reaching a ratio of 0.8, the settling time exhibited stability. The *S_f_* determination experiments on the CCC columns yielded an *S_f_* of 34.4% for ACN/THF/DEHPA/aqueous saturated NaCl (2:2:0.4:3, *v*/*v*) and an *S_f_* of 45.8% for ACN/THF/DEHPA/aqueous saturated NaCl (2:2:0.8:3, *v*/*v*). Therefore, ACN/THF/DEHPA/aqueous saturated NaCl (2:2:0.8:3, *v*/*v*) was employed for the separation of *G. biloba* seed extract.

**Figure 3:**
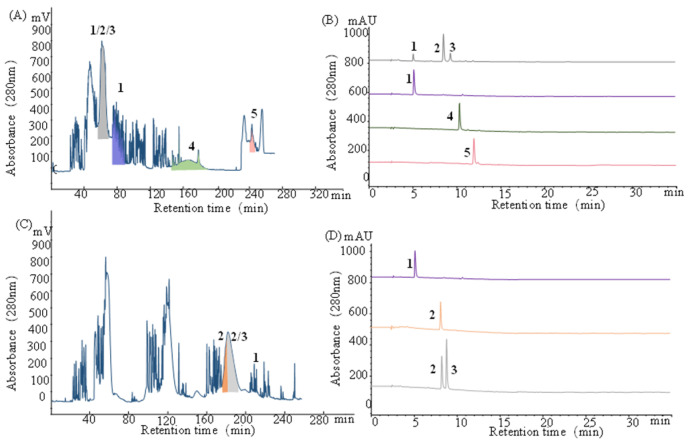
Two-step CCC separation of *G. biloba* seeds. (**A**) CCC profile using the ACN/THF/DEHPA/aqueous saturated NaCl (2:2:0.8:3, *v*/*v*) solvent system. The upper phase is the stationary phase, while the lower phase is the mobile phase. Rotation speed: 1000 rpm, flow rate: 4 mL/min, *S_f_*: 45.8%. (**B**) HPLC analysis of the fractions of CCC in (**A**). (**C**) Three rounds of inner-recycling chromatographic procedures for a fraction in (**A**) (marked in gray color). Solvent system: ACN/THF/DEHPA/aqueous saturated NaCl (2:2:0.8:3, *v*/*v*), rotation speed: 1000 rpm, flow rate: 4 mL/min, *S_f_*: 46.0%. (**D**) HPLC analysis of the fractions of CCC in (**C**). The components numbered **1** to **5** represent the five primary elements identified in the extract.

As shown in Figure 3A,B, compounds **1**, **4**, and **5** were effectively purified from the methanol extract in a one-step CCC separation, yielding HPLC purities (via the area normalization method) of 88.7%, 92.4%, and 90.2%, as determined by HPLC analysis. However, the co-elution of compounds **2** and **3** with a portion of compound **1** was observed. The inner-recycling mode has been demonstrated as an effective separation strategy for compounds with similar *K* values [27,48]. Therefore, the utilization of the inner-recycling mode was used for further separation. As shown in Figure 3C,D, through three rounds of inner-recycling chromatographic procedures, compounds **1** and **2** were purified, while compound **3** was effectively enriched. The subsequent purification of compound **3** was carried out using semi-preparative HPLC (10 mm i.d. × 250 mm, 5 μm, YMC, Kyoto, Japan) with an isocratic elution mode at a flow rate of 2 mL/min and a mobile phase consisting of a 20% methanol solution.

However, the process involved the use of ACN, THF, and DEHPA, which are environmentally unfriendly solvents. DEHPA, as a ligand used to enhance separation efficiency, is challenging to replace due to its unique properties. In subsequent studies, efforts will focus on screening greener solvents to substitute for ACN and THF. In contrast, ATPSs are preferred for separating polar components due to their environmental friendliness. If separation efficiency with an ATPS does not meet requirements, this alternative system can still serve as a viable option. For the purification of the indole alkaloid N-glycoside from Ginkgo fruit, Yea Jung Choi et al. utilized a five-step process that included solvent partitioning; HP 20 column chromatography; silica gel column chromatography; and preparative as well as semi-preparative reversed-phase HPLC using a gradient solvent system comprising hexane, dichloromethane, ethyl acetate, n-butanol, and methanol [32]. In the study by Jin-Tang Cheng et al., repeated column chromatography was also required, utilizing solvents such as ACN, dichloromethane, chloroform, and methanol [31]. Compared to these methods, the method developed in this work involves fewer steps and less solvent usage, making it more economical and efficient.

### 2.4. Identification of the Isolated Compounds

**Figure 4:**
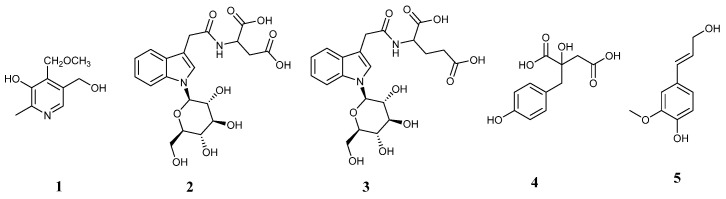
Structures of the compounds separated from *G. biloba* seeds.

The five compounds were identified through the utilization of high-resolution MS and NMR analysis (Supplementary Materials), with the structures depicted in Figure 4.

Compound **1** showed a formula ofC_9_H_14_O_3_N (*m/z* 184.09676 [M+H]^+^, calculated for *m/z* 184.09682). ^1^H NMR spectrum (400 MHz, DMSO-*d6*): δ 7.96 (1H, s, H-6), 4.55 (2H, s, H-4′), 4.51 (2H, s, H-5′), 3.27 (3H, s, H-4′-OCH_3_), 2.36 (3H, s, H-2′). ^13^C NMR (100 MHz, DMSO-*d6*): δ 19.7 (C-2′), 57.8 (C-4′-OCH_3_), 58.6 (C-5′), 64.6 (C-4′), 128.9 (C-5), 134.6 (C-4), 139.1 (C-6), 146.1 (C-2), 149.2 (C-3). When compared with the data given in reference [49], it was identified as 4′-O-methylpyridoxine.

Compounds **2** showed a formula of C_20_H_23_O_10_N_2_ (*m/z* 451.13574 [M−H]^−^, calculated for *m/z* 451.13472). ^1^H NMR (400 MHz, DMSO-*d6*): δ 7.54 (1H, d, *J* = 7.8 Hz, H-4), 7.50 (1H, d, *J* = 8.3 Hz, H-7), 7.32 (1H, s, H-2), 7.11 (1H, t, *J* = 7.2 Hz, H-6), 7.01 (1H, t, *J* = 7.3 Hz, H-5), 5.38 (1H, d, *J* = 9.1 Hz, H-1′), 4.55 (1H, m, H-10), 3.70 (1H, m, H-2′), 3.68 (1H, m, H-6′β), 3.56 (2H, s, H-8), 3.55 (1H, m, H-5′), 3.46 (1H, m, H-6′α), 3.42 (1H, m, H-3′), 3.25 (1H, m, H-4′), 2.73 (1H, m, H-11β), 2.62 (1H, m, H-11α). ^13^C NMR (100 MHz, DMSO-*d6*): δ 32.1(C-8), 36.1 (C-11), 48.8 (C-10), 60.9 (C-6′), 69.9 (C-4′), 71.9 (C-2′), 77.6 (C-3′), 79.4 (C-5′), 84.4 (C-1′), 109.3 (C-3), 110.5 (C-7), 119.1 (C-4), 119.2 (C-5), 121.3 (C-6), 124.5 (C-2), 128.1 (C-3α), 136.5 (C-7α), 170.4 (C-9), 171.8 (C-10α), 172.5 (C-11α). When compared with the data given in reference [31], it was identified as ginkgoside B.

Compounds **3** showed a formula of C_21_H_25_O_10_N_2_ (*m/z* 465.15109 [M−H]^−^, calculated for *m/z* 465.15037). ^1^H NMR (400 MHz, DMSO-*d6*): δ 7.55 (1H, d, *J* = 7.8 Hz, H-4), 7.50 (1H, d, *J* = 8.3 Hz, H-7), 7.32 (1H, s, H-2), 7.12 (1H, t, *J* = 6.7 Hz, H-6), 7.01 (1H, t, *J* = 7.4 Hz, H-5), 5.39 (1H, d, *J* = 9.0 Hz, H-1′), 4.22 (1H, m, H-10), 3.71 (1H, m, H-2′), 3.68 (1H, m, H-6′β), 3.58 (2H, s, H-8), 3.55 (1H, m, H-5′), 3.46 (1H, m, H-6′α), 3.41 (1H, m, H-3′), 3.25 (1H, m, H-4′), 2.31 (1H, m, H-11), 1.98 (1H, m, H-12β), 1.81 (1H, m, H-12α). ^13^C NMR (100 MHz, DMSO-*d6*): δ 26.4 (C-12), 30.1 (C-11), 32.0 (C-8), 51.3 (C-10), 60.9 (C-6′), 69.9 (C-4′), 71.9 (C-2′), 77.6 (C-3′), 79.4 (C-5′), 84.4 (C-1′), 109.4 (C-3), 110.5 (C-7), 119.0 (C-4), 119.2 (C-5), 121.3 (C-6), 124.5 (C-2), 128.1 (C-3α), 136.5 (C-7α), 170.6 (C-9), 173.5 (C-10α), 173.8 (C-12α). When compared with the data given in reference [31], it was identified as ginkgoside A.

Compound **4** showed a formula of C_11_H_11_O_6_ (*m/z* 239.05571 [M−H]^−^, calculated for *m/z* 239.05501). ^1^H NMR (400 MHz, DMSO-*d6*): δ 6.99 (2H, d, *J* = 8.4 Hz, H-2′, 6′), 6.63 (2H, d, *J* = 8.4 Hz, H-3′, 5′), 2.81 (1H, d, *J* = 9.6 Hz, H-5β), 2.77 (1H, d, *J* = 9.6 Hz, H-5α), 2.71 (1H, d, *J* = 15.8 Hz, H-3β), 2.35 (1H, d, *J* = 15.8 Hz, H-3α). ^13^C NMR (100 MHz, DMSO-*d6*): δ 42.5 (C-5), 43.7 (C-3), 74.9 (C-2), 114.6 (C-3′, 5′), 126.2 (C-1′), 131.4 (C-2′, 6′), 156.0 (C-4′), 171.7 (C-3), 175.7 (C-1). When compared with the data given in reference [50], it was identified as 2-(4-hydroxybenzyl) malic acid.

Compound **5** showed a formula of C_10_H_11_O_3_ (*m/z* 179.07053 [M−H]^−^, calculated for *m/z* 179.07027). ^1^H NMR (400 MHz, DMSO-*d6*): δ 6.99 (2H, d, *J* = 1.7 Hz, H-5), 6.79 (1H, dd, *J* = 8.1, 1.7 Hz, H-9), 6.79 (1H, d, *J* = 8.1 Hz, H-8), 6.41 (1H, d, *J* = 15.9 Hz, H-3), 6.17 (1H, dt, *J* = 15.9, 5.4 Hz, H-2), 4.07 (2H, dd, *J* = 5.4, 1.2 Hz, H-1), 3.77 (3H, s, -OCH_3_). ^13^C NMR (100 MHz, DMSO-*d6*): δ 55.5 (--OCH_3_), 61.7 (C-1), 109.7 (C-5), 115.5 (C-8), 119.4 (C-9), 127.5 (C-2), 128.4 (C-4), 129.0 (C-3), 146.2 (C-7), 147.7 (C-6). When compared with the data given in reference [51], it was identified as coniferyl alcohol.

## 3. Materials and Methods

### 3.1. Chemicals and Samples

Analytical-grade reagents used for CCC included di-(2-ethylhexyl) phosphoric acid (DEHPA, CAS: 298-07-7), polyethylene glycol 1000 (PEG-1000, CAS: 25322-68-3) (Beijing InnoChem Science & Technology Co., Ltd., Beijing, China), acetonitrile (ACN, CAS: 75-05-8), n-butanol (n-BuOH, CAS: 71-36-3), isopropanol (IPA, CAS: 67-63-0), ethanol (CAS: 64-17-5), tetrahydrofuran (THF, CAS: 109-99-9), methanol (M, CAS: 67-56-1), triethylamine (TEA, CAS: 121-44-8), hydrochloric acid (HCl, CAS: 7647-01-0), ammonium sulfate [(NH_4_)_2_SO_4_, CAS: 7783-20-2], and sodium chloride (NaCl, CAS: 7647-14-5) (Chron Chemicals Co., Ltd., Chengdu, China). Chromatographic-grade methanol and trifluoroacetic acid (TFA, CAS: 76-05-1) (J&K Scientific Ltd.) were used for HPLC analysis. (Beijing, China). The water used was bottled purified water (CAS: 7732-18-5, Wahaha Group Co., Ltd., Hangzhou, China). *G. biloba* seeds were procured from Xuzhou, Jiangsu, China.

### 3.2. CCC System

The study employed an OptiChrome-300PLUS HSCCC, manufactured by Jiangyin Counter Current Technology Co., Ltd. (Jiangyin, China) and equipped with two multilayer coil separation columns. These columns were connected in series and had a combined volume of 300 mL. The tubing had an inner diameter (i.d.) of 2.1 mm, and the sample loop contained 20 mL. The revolution radius was set at 5 cm, while the *β* values of the multilayer coils ranged from 0.6 at the internal terminal to 0.8 at the external terminal. A speed controller regulated the rotational speed of the apparatus within a range of 0 to 1200 rpm. Additionally, two LC-3000 metering pumps, a UV 2000D spectrometer, a CXTH-3000 workstation (Beijing Tong Heng Innovation Technology Co., Ltd., Beijing, China), and a BSZ-100 fraction collector were also incorporated.

### 3.3. Sample Preparation

The fresh *G. biloba* seeds were crushed, and 6 kg of particles were extracted using a dip extraction method. Methanol was employed as the solvent for extraction. Two rounds of extraction were conducted, with each process utilizing 10 L of methanol over a duration of one week. The filtrated solvent was combined and subjected to solvent evaporation, resulting in an approximate yield of 50 g of crude sample.

### 3.4. Solvent System Selection and CCC Separation

#### 3.4.1. Solvent System Selection

The solvent systems for CCCS were selected based on the *K* values of the major compounds in the crude extract. A solvent system was deemed suitable if it provided *K* values between 0.25 and 4 for most of the primary constituents [33]. Solvent systems incorporating various solvents were initially configured. Systems that exhibited no stratification, severe emulsification upon sample addition, or significant volume discrepancies between the upper and lower phases were initially excluded. The *K* values of the primary components in the crude extract were determined using a modified version of the “HPLC-shake flask” method [52]. Briefly, the sample (∼10 mg) was dissolved in equal volumes (800 μL) of the upper and lower phases of a selected solvent system, followed by subsequent HPLC analysis. A_1_ and A_2_ represent the peak areas detected in the upper phase and lower phase, respectively. *K* was calculated using Equation (1) based on A_1_ and A_2_.

*K*=*A*_1_/*A*_2_ (1)

The HPLC analysis was conducted on an Agilent 1100 system (Santa Clara, CA, USA) comprising a G1379A degasser, a G1311A quaternary pump, a G1367A well plate sampler, a G1316A column oven, a G1315B diode array detector, and an Agilent ChemStation, using a YMC-C_18_ column (4.6 mm i.d. × 150 mm, 5 μm, YMC, Kyoto, Japan) in a gradient elution mode. The mobile phases consisted of an aqueous solution of 0.03% TFA (A) and methanol (B). The flow rate was set at 0.8 mL/min. The elution procedure proceeded as follows: from 0 to 25 min, the proportion of B increased from 10% to 100%, and then it remained at 100% from minute 25 to minute 35. A wavelength of 280 nm was selected for monitoring the effluent.

#### 3.4.2. CCCS

The CCC separations were performed in a “head-to-tail” mode, where the upper phase was employed as the stationary phase and initially introduced into the CCC columns. Subsequently, the apparatus was activated, which was followed by pumping the lower phase at a flow rate of 2 or 4 mL/min. Once dynamic equilibrium was achieved, *S_f_* was calculated according to literature descriptions [4,53]. Subsequently, the sample solutions were introduced into the CCC through the injection valve. The effluent was monitored using a UV detector set at 280 nm wavelength. The separation process was conducted at a temperature of 30 °C.

#### 3.4.3. MS and NMR Analysis

The NMR analysis was performed on a Bruker Avance Neo 400 NMR spectrometer (Bruker, Fallanden, Switzerlad). NMR data were collected at 25 °C, operating at 400 MHz for ^1^H and 100 MHz for ^13^C. The solvent used for all samples was DMSO-*d6*, and the chemical shifts were referenced to that of TMS, which served as the internal standard. For acquiring ^1^H NMR spectra, 16 scans were collected over a spectral width of 0–20.48 ppm (8196.72 Hz), with an acquisition time of 4.00 s, a relaxation delay of 1 s, and an FID resolution of 0.25 Hz. To acquire ^13^C NMR spectra, 1024 scans were collected over a spectral width of 0–236.62 ppm (23.81 KHz), with an acquisition time of 1.38 s, a relaxation delay of 2 s, and an FID resolution of 0.73 Hz.

The MS analysis was performed with a Q-Exactive Focus hybrid quadrupole-orbitrap mass spectrometer (Thermo Fisher Scientific Inc., Bremen, Germany). The mass spectrometer was operated in both positive and negative electrospray ionization (ESI) mode over a mass range of *m*/*z* 100–1500. The other parameters were set as follows: spray voltage at 3.3 kV, sheath gas flow rate at 35 L/min, auxiliary gas flow rate at 5 L/min, capillary temperature at 320 °C, and S-lens RF level at 50 V. Scan modes included full MS with a resolution of 70,000. Automatic gain control was adjusted to maintain an intensity target of 1 × 106 for MS. The maximum injection time (IT) for full MS analysis was set to 100 ms. Dynamic exclusion was implemented, with the duration of exclusion set to be within a period of six seconds. Data handling was performed using Xcalibur™4.4 software provided by Thermo Fisher.

## 4. Conclusions

Polar components such as polysaccharides and glucoside have garnered increasing attention due to their significant biological activities. However, their separation remains a formidable challenge, with column chromatography often necessitating laborious and repetitive purification processes. CCCS based on ATPSs frequently offers an advantageous alternative. When conventional ATPSs fail to meet the required standards, the ACN/THF/DEHPA/saturated aqueous NaCl solvent system emerges as a viable and efficient solution.

The development of this system has a significant positive impact on the discovery and utilization of polar constituents from *G. biloba* seeds, particularly the indole alkaloid N-glycosides, which are uncommon in natural products. Previous studies have demonstrated their low cytotoxicity along with pronounced anti-inflammatory activities and anti-skin-aging effects. Future research is likely to uncover additional therapeutic activities, positioning these compounds as promising candidates for development in medicine, food, and cosmetics. They may emerge as a novel research focus following the standardized extraction of *G. biloba* leaves.

## Figures and Tables

**Table 1:** *K* values of the main compounds in different solvent systems.

Compounds	Solvent Systems						
	n-BuOH/H_2_O	n-BuOH/H_2_O (10 mM HCl)	n-BuOH/H_2_O (10 mM TEA)	ACN/Aqueous Saturated NaCl	IPA/Aqueous Saturated NaCl	15% PEG-1000/15% (NH_4_)_2_SO_4_	18% PEG-1000/15% (NH_4_)_2_SO_4_
1	1.20	1.30	0.31	0.27	5.50	1.29	1.47
2	\	\	\	\	\	1.62	1.90
3	\	\	\	\	\	1.94	2.56
4	\	\	\	\	\	1.98	2.66
5	/	/	/	/	6.05	5.96	13.37

The symbols “\” and “/” indicate that the *K* values were insufficiently small and excessively large, respectively.

**Table 2:** *K* values of the main compounds in ACN/THF/DEHPA/aqueous saturated NaCl.

Compounds	ACN/THF/D2EHPA/Aqueous Saturated NaCl (2:2:x:3, *v*/*v*)					
	0	0.4 (58.6 s) *	0.6 (51.1 s)	0.8 (43.9 s)	1 (43.2 s)	1.2 (42.7 s)
1	1.35	1.94	2.13	2.37	2.63	2.49
2	\	0.93	0.84	0.73	0.64	0.51
3	\	1.26	1.13	0.99	0.87	0.74
4	0.34	3.88	5.51	4.8	4.43	3.6
5	/	7.02	9.99	8.84	8.8	7.05

“*” the settling time measured in a test tube; “\” and “/” indicate that the K values were insufficiently small and excessively large, respectively.

## Data Availability

The data are available from the corresponding author on reasonable request.

## Supplementary Materials

The following supporting information can be downloaded at: https://www.mdpi.com/article/10.3390/molecules30020409/s1.
