# Supplementary material for: Screening of Solvent Systems for Countercurrent Chromatography Separation of Polar Constituents from Ginkgo biloba L. Seeds
Source: Molecules. 2025 Jan 19;30(2):409. doi: 10.3390/molecules30020409 (PMC11767392; doi:10.3390/molecules30020409)
Supplement: Supplementary file 1 [file molecules-30-00409-s001.zip › molecules-3392139-supplementary.pdf]

Article

Screening of solvent systems for countercurrent chromatography separation of polar constituents from *Ginkgo biloba* L. seeds

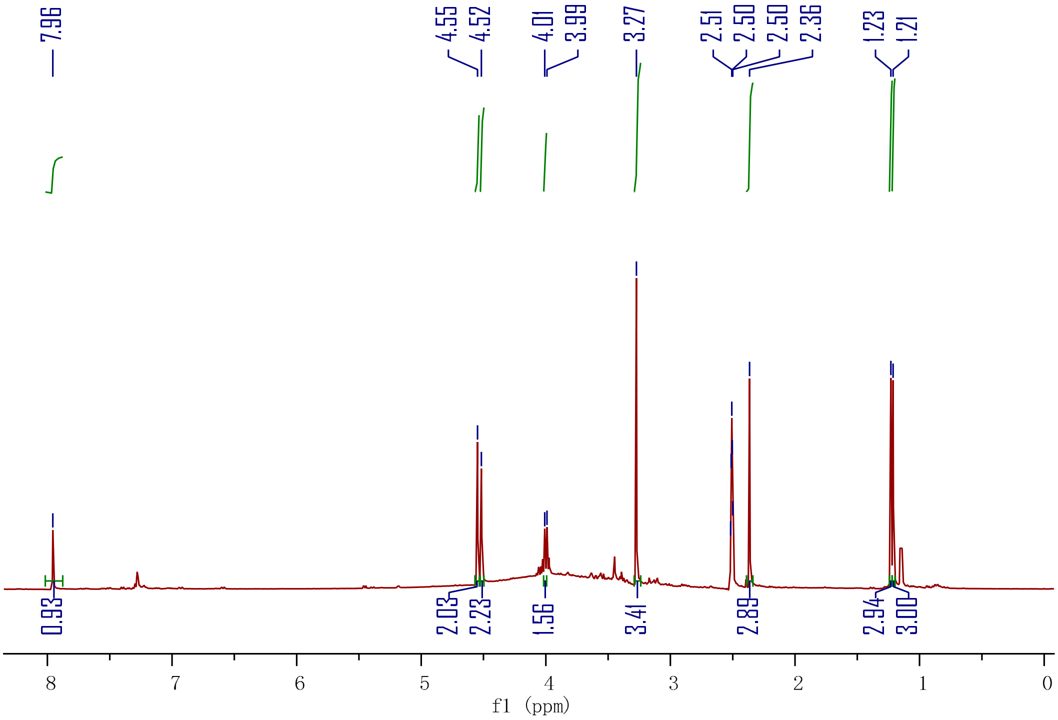

Figure S1. ^{1}H NMR analysis of compound **1**.

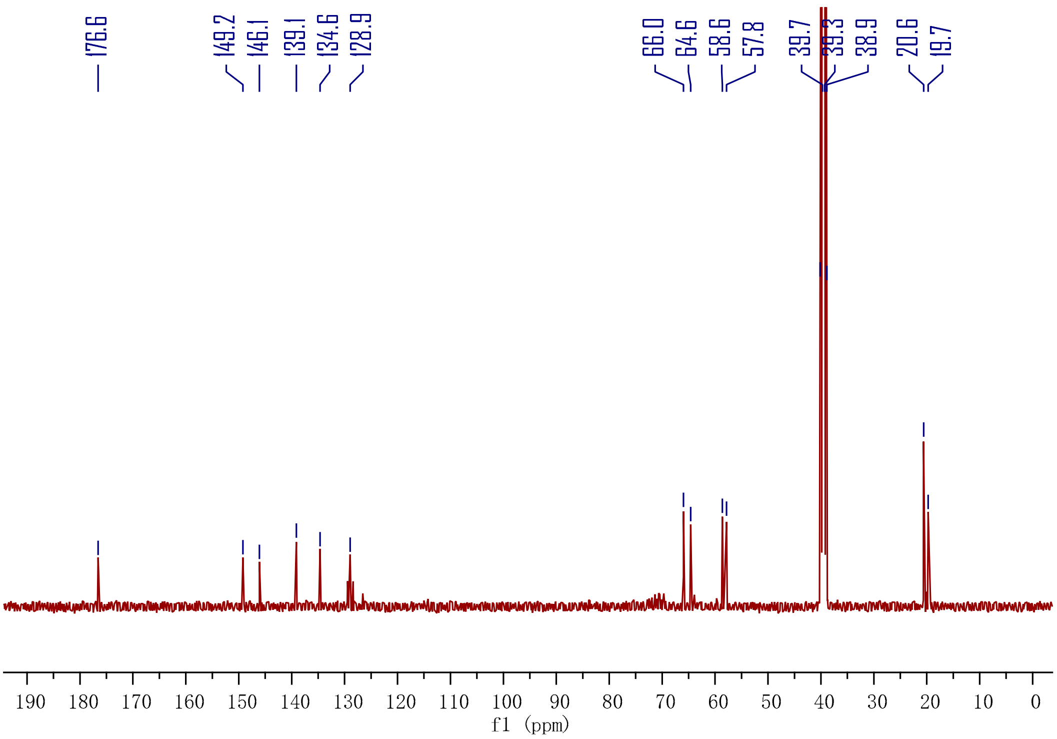

Figure S2. ^{13}C NMR analysis of compound **1**

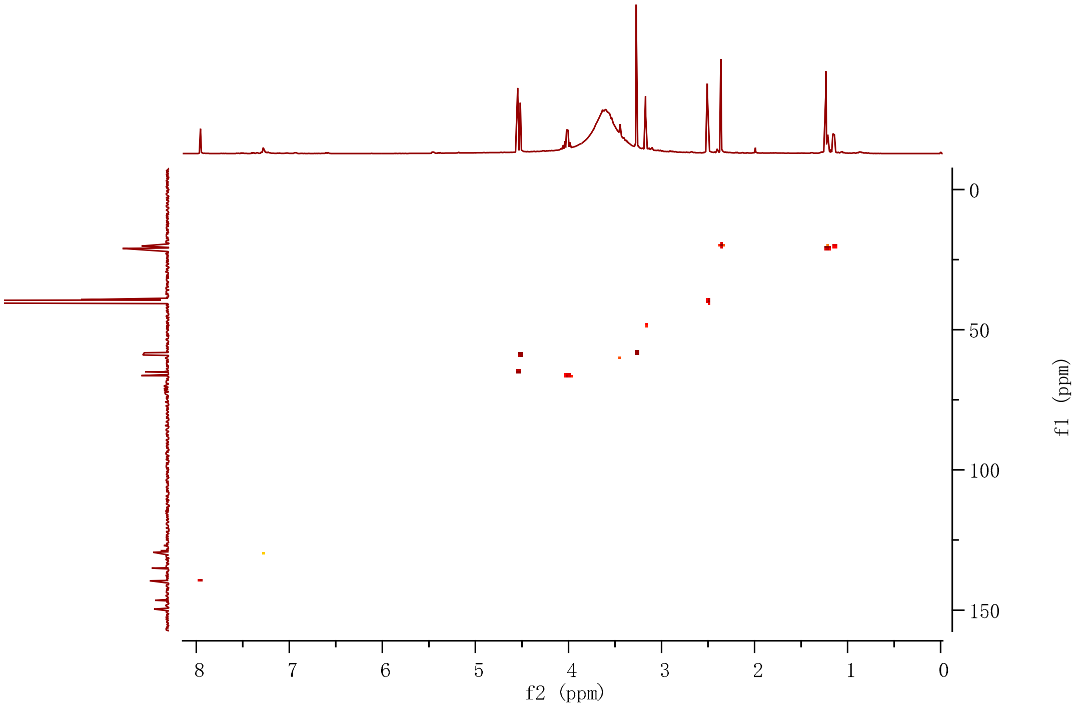

Figure S3. HMQC NMR analysis of compound **1**.

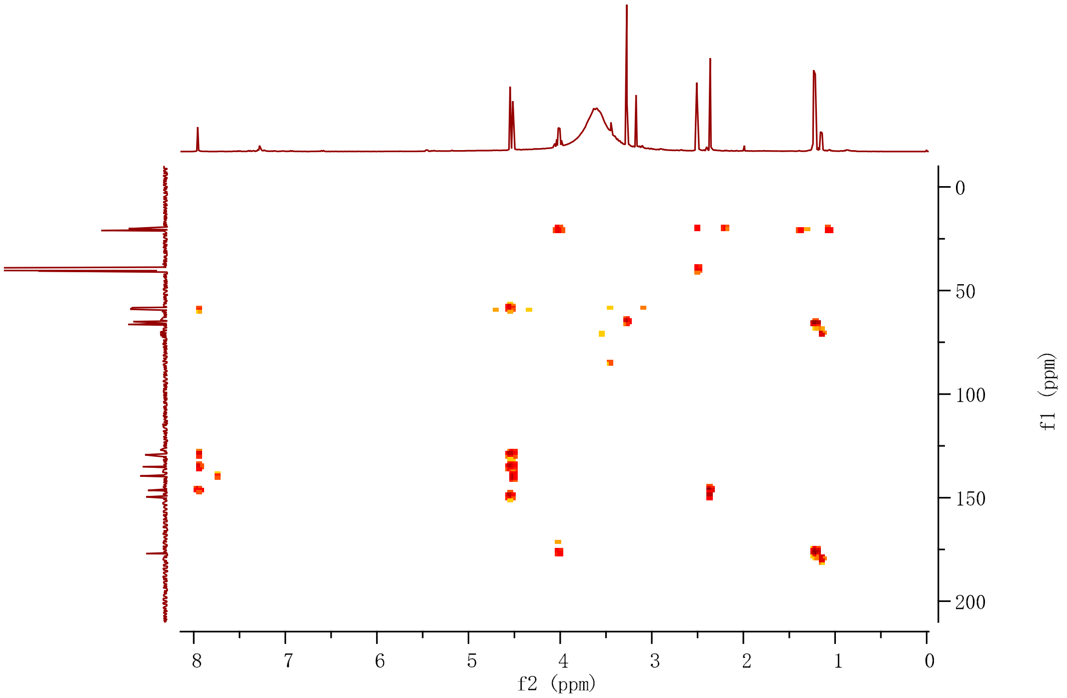

Figure S4. HMBC NMR analysis of compound **1**.

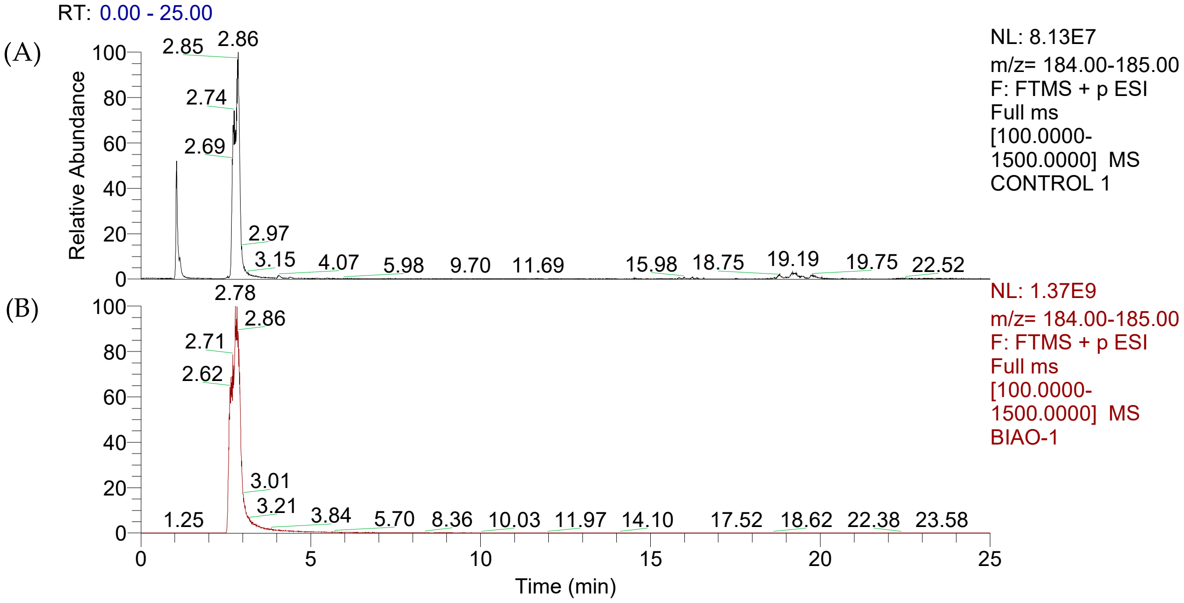

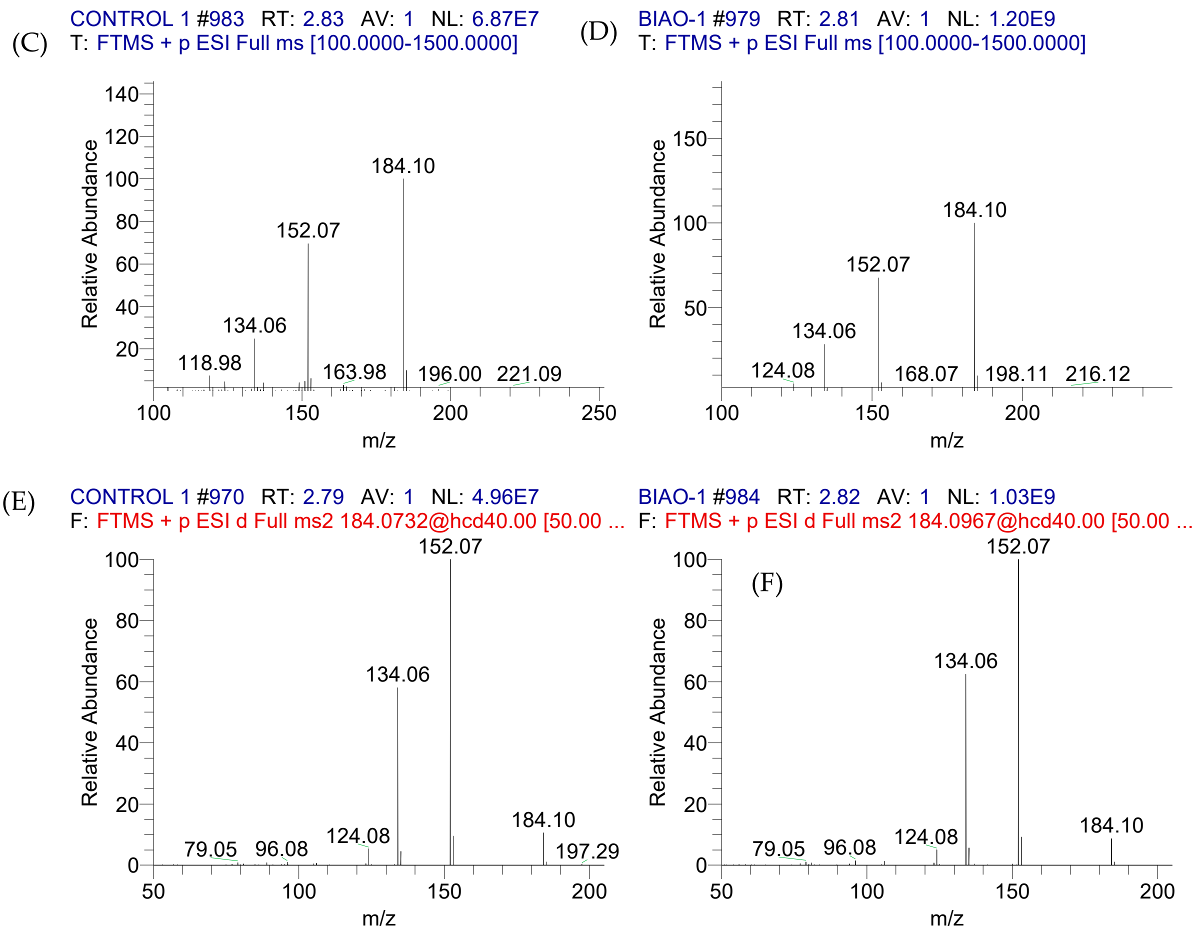

Figure S5. UHPLC-HRMS/MS analysis of compound **1** (A, C, E) and a standard of 4′-O-methylpyridoxine (B, D, F).

Some signals observed in the ^{1}H NMR (δ 1.21, 1.23, and 4.00) and ^{13}C NMR (δ 20.6, 66.0, and 176.6) spectra are attributed to impurities. HMQC and HSQC analysis revealed no correlations between these signals and any other peaks. To further confirm this, a standard sample of 4′-O-methylpyridoxine was obtained, and its UHPLC-HRMS/MS profile was compared with that of compound **1**. The comparison showed that both the retention times and the primary and secondary mass spectra were consistent.

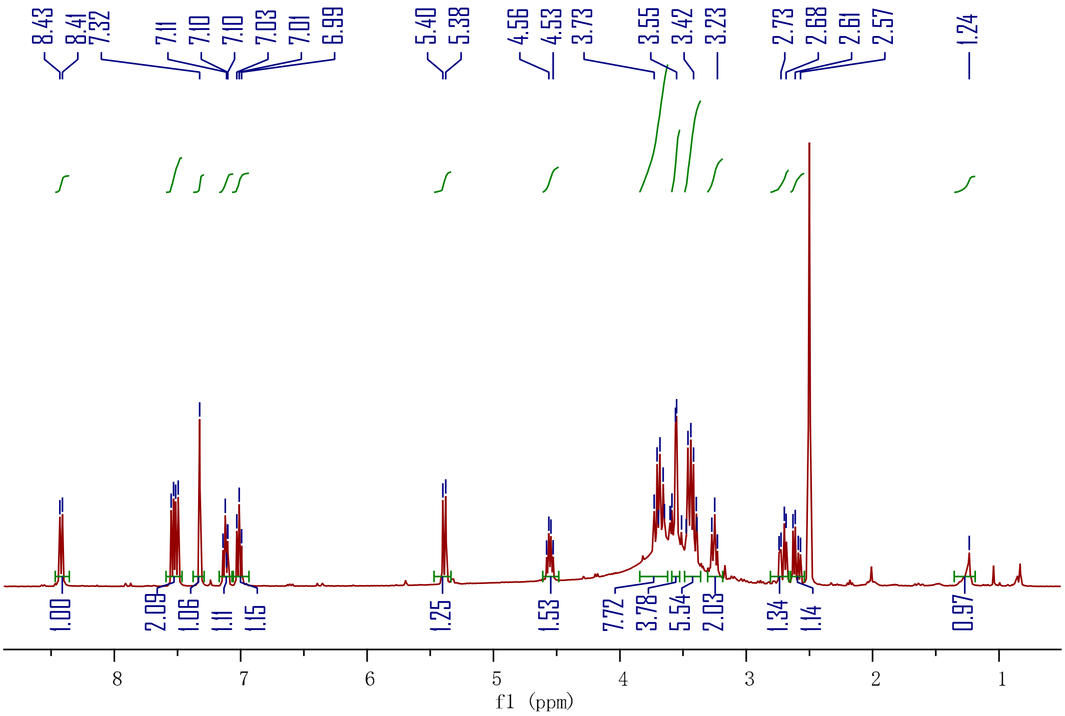

Figure S6. ^{1}H NMR analysis of compound **2.**

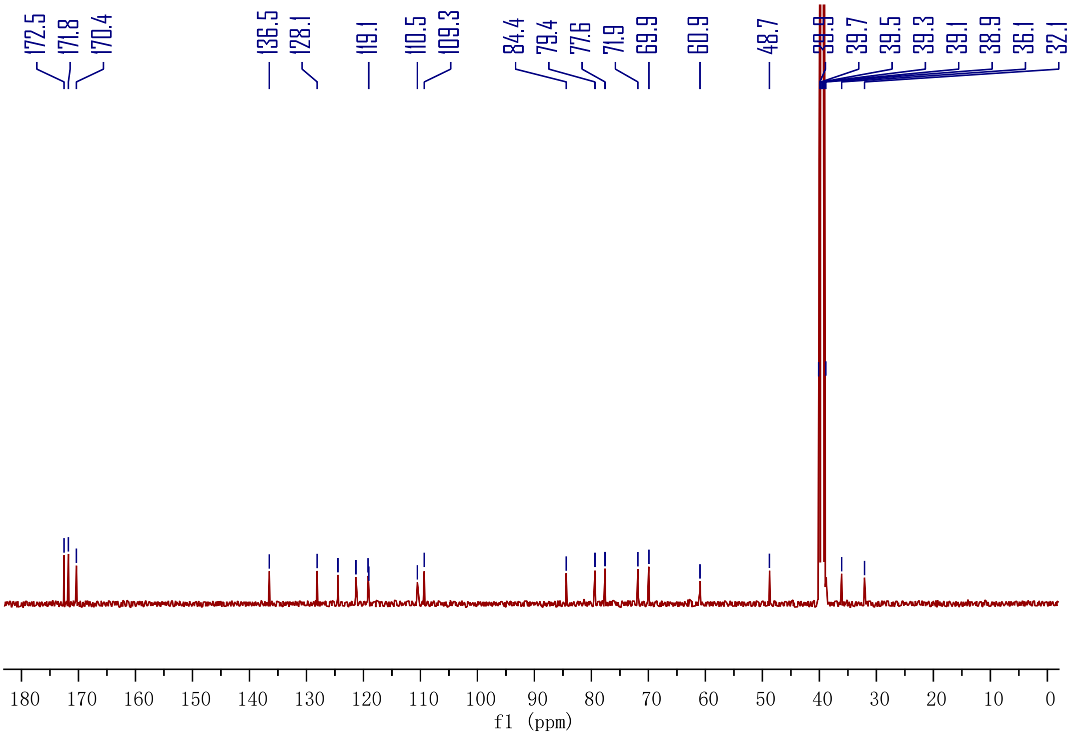

Figure S7. ^{13}C NMR analysis of compound **2.**

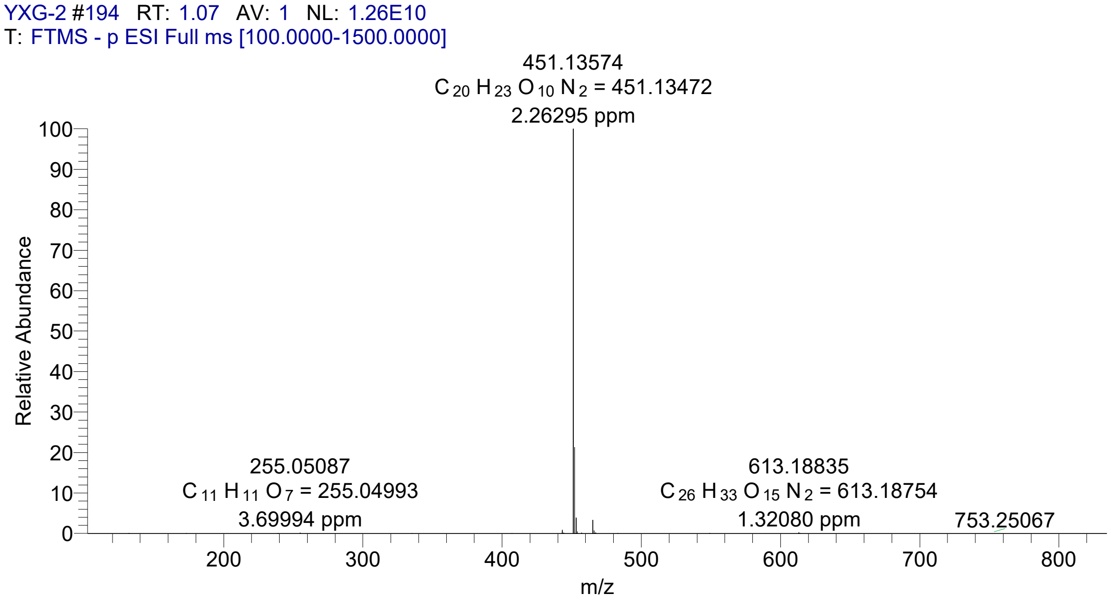

Figure S8. HRMS analysis of compound **2.**

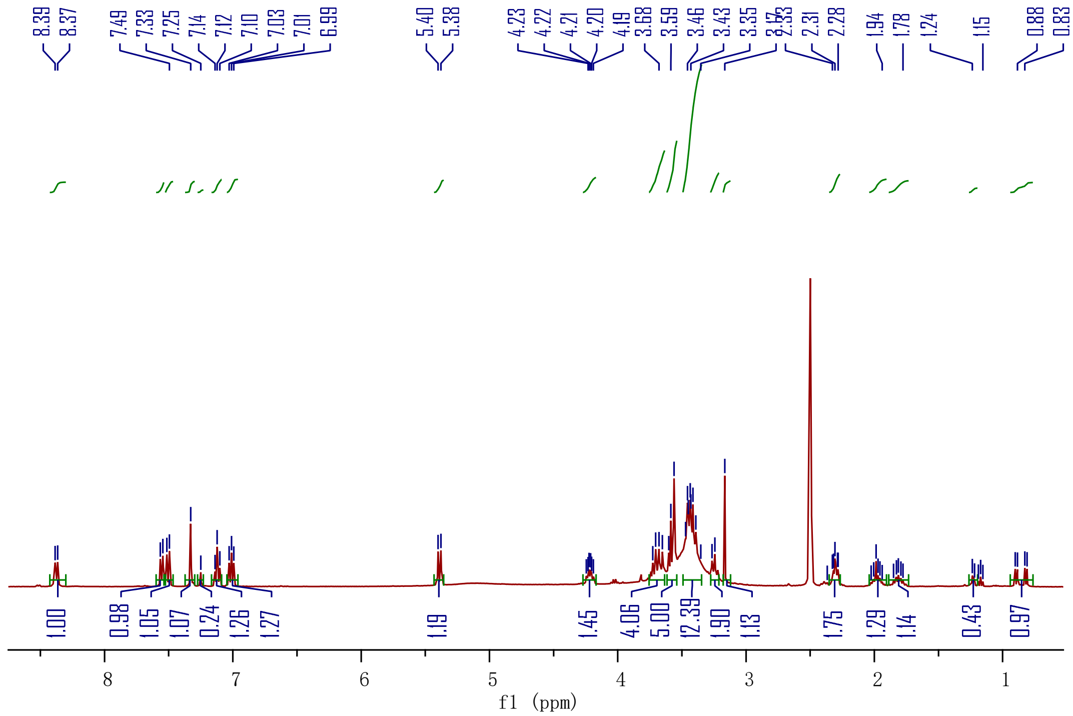

Figure S9. ^{1}H NMR analysis of compound **3.**

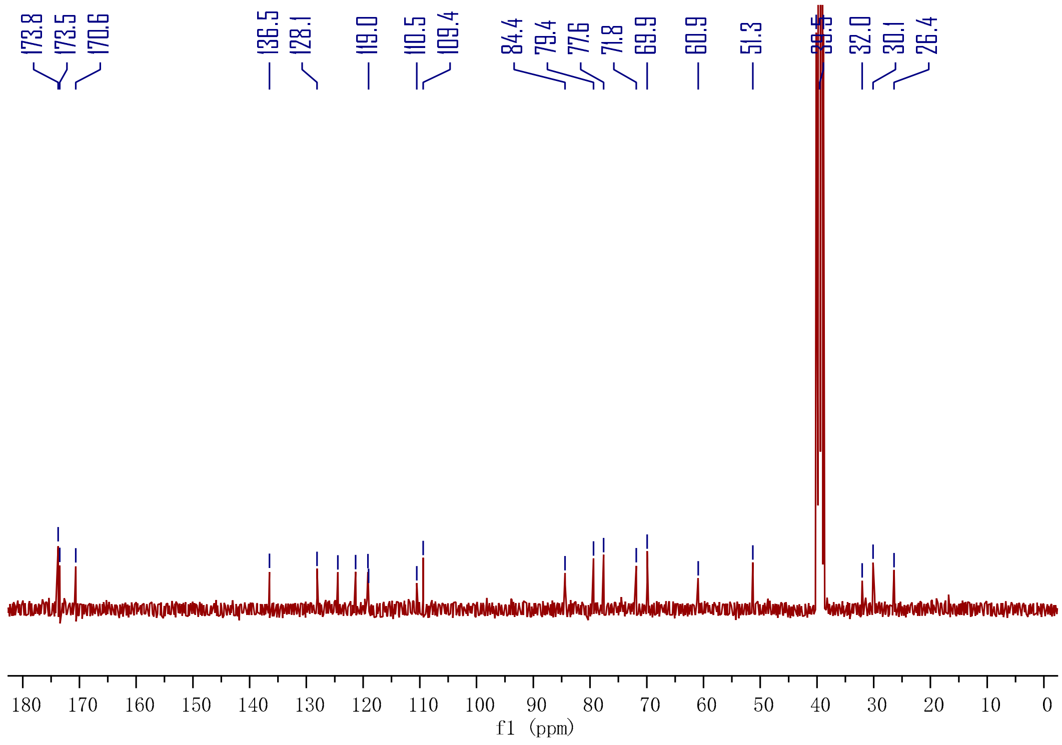

Figure S10. ^{13}C NMR analysis of compound **3.**

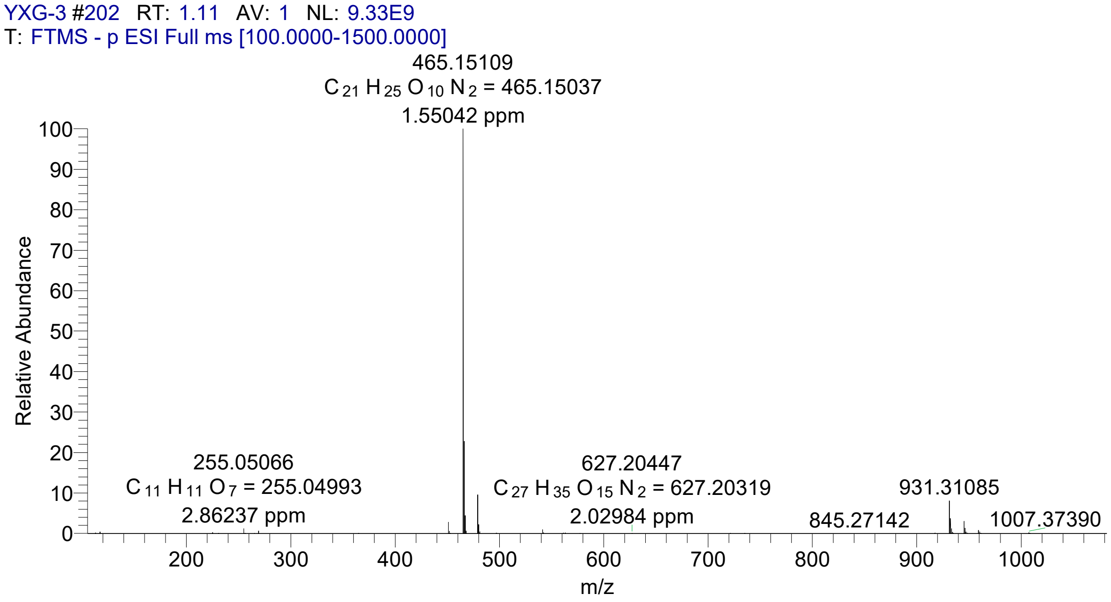

Figure S11. HRMS analysis of compound **3.**

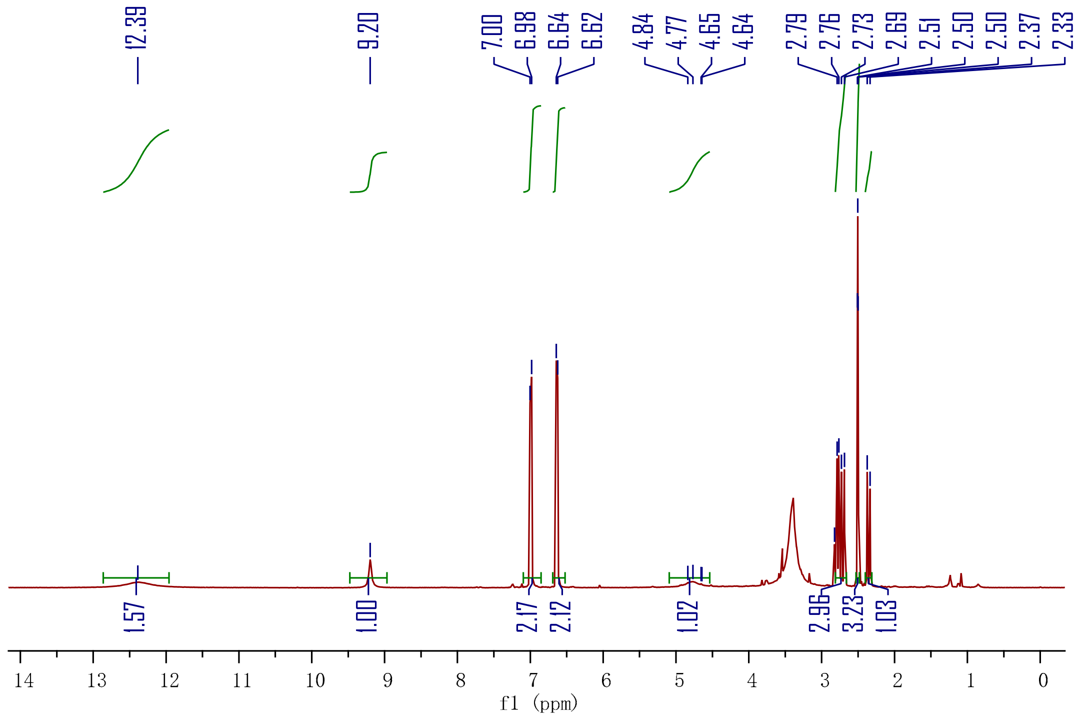

Figure S12. ^{1}H NMR analysis of compound **4.**

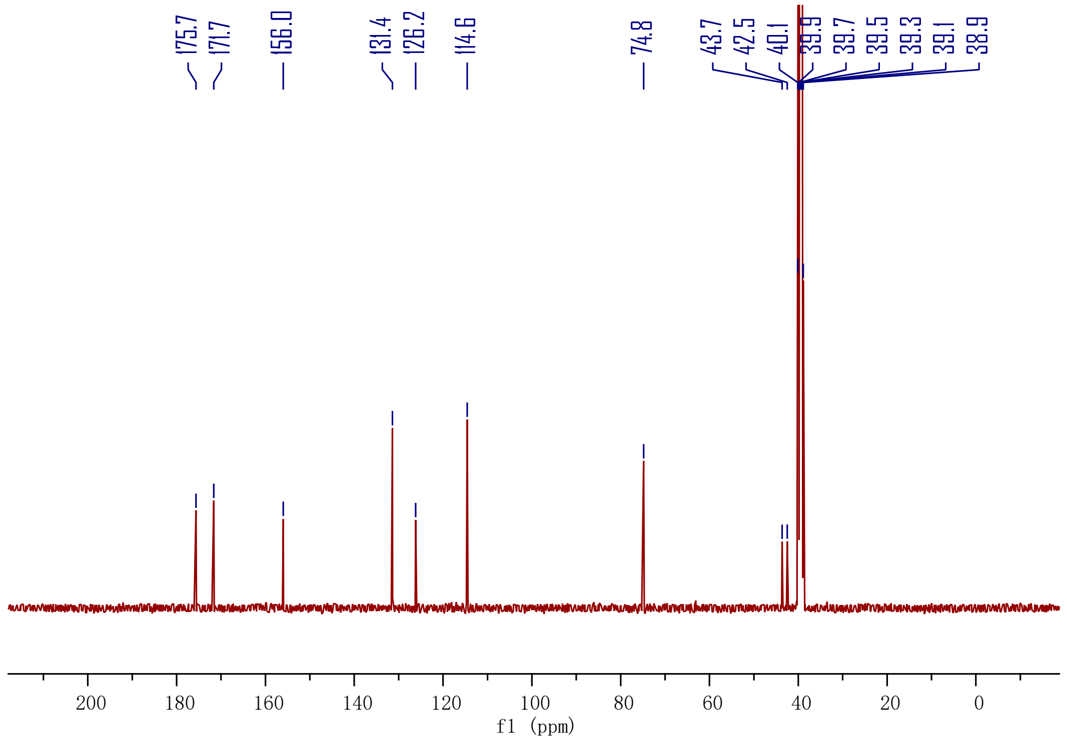

Figure S13. ^{13}C NMR analysis of compound **4.**

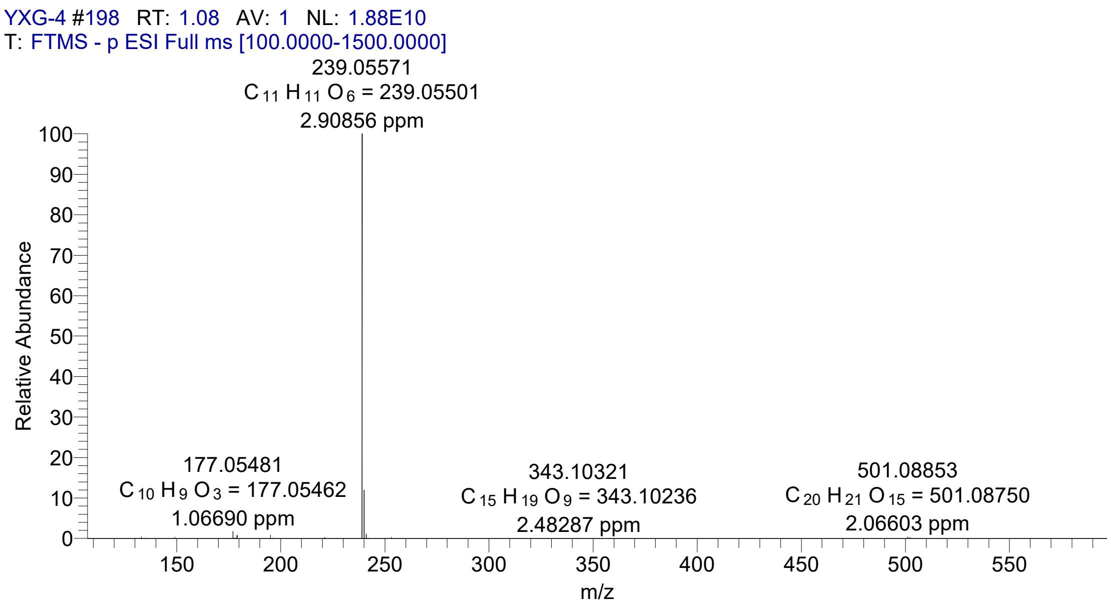

Figure S14. HRMS analysis of compound **4.**

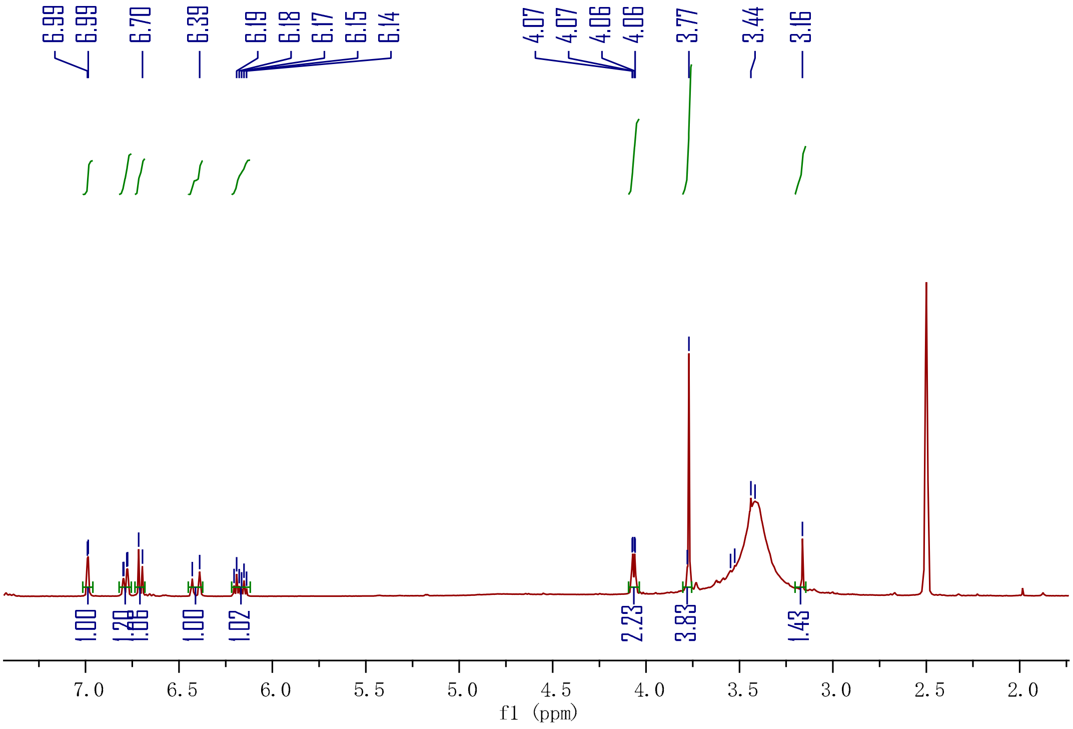

Figure S15. ^{1}H NMR analysis of compound **5.**

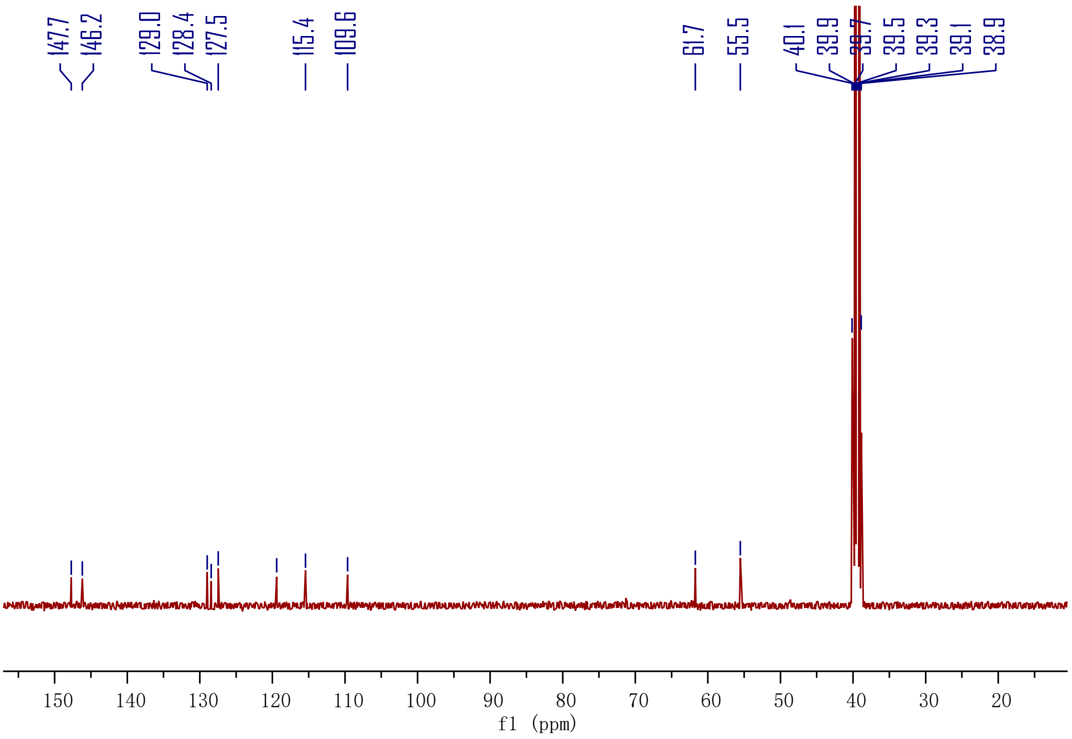

Figure S16. ^{13}C NMR analysis of compound **5.**

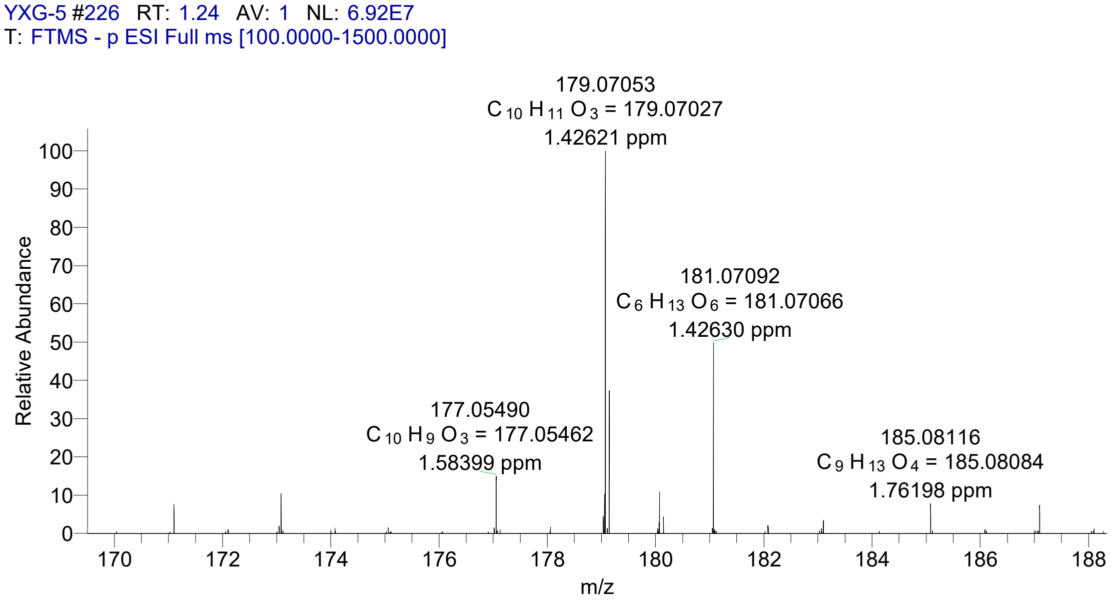

Figure S17. HRMS analysis of compound **5.**
